# From intramuscular to nasal: unleashing the potential of nasal spray vaccines against coronavirus disease 2019

**DOI:** 10.1002/cti2.1514

**Published:** 2024-05-20

**Authors:** Ge Jin, Runze Wang, Yi Jin, Yingqiu Song, Tianlu Wang

**Affiliations:** ^1^ Faculty of Medicine Dalian University of Technology Dalian Liaoning China; ^2^ Department of Radiotherapy Cancer Hospital of China Medical University, Liaoning Cancer Hospital and Institute Shenyang Liaoning China; ^3^ Department of Breast Surgery Liaoning Cancer Hospital and Institute Shenyang Liaoning China; ^4^ Department of Radiotherapy Cancer Hospital of Dalian University of Technology Dalian Liaoning China

**Keywords:** COVID‐19, immunity, nasal cavity, SARS‐CoV‐2, vaccine

## Abstract

Coronavirus disease 2019, caused by severe acute respiratory syndrome coronavirus 2 (SARS‐CoV‐2), has affected 700 million people worldwide since its outbreak in 2019. The current pandemic strains, including Omicron and its large subvariant series, exhibit strong transmission and stealth. After entering the human body, the virus first infects nasal epithelial cells and invades host cells through the angiotensin‐converting enzyme 2 receptor and transmembrane serine protease 2 on the host cell surface. The nasal cavity is an important body part that protects against the virus. Immunisation of the nasal mucosa produces immunoglobulin A antibodies that effectively neutralise viruses. Saline nasal irrigation, a type of physical therapy, can reduce the viral load in the nasal cavity and prevent viral infections to some extent. As a commonly used means to fight SARS‐CoV‐2, the intramuscular (IM) vaccine can induce the human body to produce a systemic immune response and immunoglobulin G antibody; however, the antibody is difficult to distribute to the nasal mucosa in time and cannot achieve a good preventive effect. Intranasal (IN) vaccines compensate for the shortcomings of IM vaccines, induce mucosal immune responses, and have a better effect in preventing infection. In this review, we discuss the nasal defence barrier, the harm caused by SARS‐CoV‐2, the mechanism of its invasion into host cells, nasal cleaning, IM vaccines and IN vaccines, and suggest increasing the development of IN vaccines, and use of IN vaccines as a supplement to IM vaccines.

## Graphical abstract

In this article, we discuss nasal mucosal immunity against SARS‐CoV‐2, the nasal cavity being an important body part that protects against the virus. Immunisation of the nasal mucosa produces immunoglobulin A antibodies that effectively neutralise viruses. Intranasal vaccine can make up for the deficiency of intramuscular vaccine in inducing nasal mucosal immunity and has a broad application prospect in the prevention and resistance of SARS‐CoV‐2.

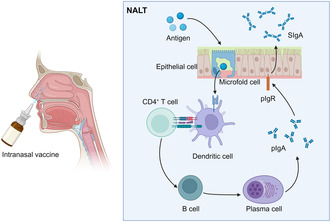

## Introduction

In 2019, the severe acute respiratory syndrome coronavirus 2 (SARS‐CoV‐2) was first detected in Wuhan, China.^1^ Since then, the virus has spread worldwide and become a global pandemic, infecting hundreds of millions of people. The symptoms of SARS‐CoV‐2 in humans are mainly respiratory; however, a few patients experience severe conditions, including breathing difficulties, shock, blood clotting disorders and organ failure.^2^ SARS‐CoV‐2 has many variants. The Omicron variant and its subvariants have become prevalent. Mutations in the virus increase its stealth and transmission. Additionally, SARS‐CoV‐2 spreads from person to person primarily through respiratory droplets or indirect contact with virus‐infected objects and subsequently touching the mouth, nose, or eyes.^3^ The nasal cavity is one of the main target organs of SARS‐CoV‐2 infection.^4^ After the virus enters the nasal cavity, it first infects nasal epithelial cells.^5^ Notably, the spike protein of SARS‐CoV‐2 binds to the angiotensin‐converting enzyme 2 (ACE2) receptor on the host cell surface and invades host cells via transmembrane serine protease 2 (TMPRSS2).^6^ As scientists broadened their research on the mechanism of SARS‐CoV‐2 infection, we identified the importance of the nasal cavity in preventing viral infections. Besides proper mask‐wearing and hand hygiene, nasal saline irrigation can be used as a topical nasal treatment to reduce viral infections.^7^

According to the World Health Organization (WHO), as of March 30, 2023, 183 vaccines have entered clinical development, and 199 vaccines have entered preclinical development.^8^ Previously developed mRNA vaccines, viral vector vaccines, inactivated vaccines, protein vaccines and other vaccines are usually used in the form of intramuscular injection, which has a high effectiveness in reducing the hospitalisation rate and mortality rate of SARS‐CoV‐2 infection.^9^ However, because SARS‐CoV‐2 has many variants and there may be many different circulating strains at different times, the validity and effectiveness of vaccine protection are greatly reduced. Moreover, the intramuscular (IM) vaccine has shortcomings in protecting the nasal cavity from SARS‐CoV‐2 infection in a timely and effective manner. It can induce an immunoglobulin G (IgG) immune response that protects the lower respiratory tract; however, most IM vaccines have a limited ability to induce immunoglobulin A (IgA) immune response that protects the upper respiratory tract.^10^, ^11^, ^12^, ^13^, ^14^, ^15^

After realising that the nasal cavity is an important link in the mechanism of viral infection, the intranasal (IN) vaccine came into being. IN vaccine can make up for the deficiency of the IM vaccine, which is difficult to induce mucosal immunity and achieve a better effect of preventing infection and preventing further transmission of virus.^16^, ^17^, ^18^ Furthermore, IN vaccines are easy to transport and store, easy to administer and painless, allowing users to self‐administer. The numerous advantages of nasal spray vaccines make them promising for future vaccine development.

## Nasal structure and defensive barrier

The human respiratory system comprises the respiratory tract and lungs. The nose, which includes the external nose, the nasal cavity and the paranasal sinuses, is the primary component of the respiratory tract. The nasal passages connect to the outer world via the nostrils and the nasopharynx through the postnasal foramen. The nasal cavity is categorised into the right and left nasal passages by the nasal septum. Each nasal cavity is split into the nasal vestibule and proper nasal cavity by the nasal threshold. The inside of the nasal vestibule is covered with nose hair to remove dust particles. Mucous membranes cover the nasal cavity and are split into the olfactory and respiratory mucous membranes. The olfactory mucosa has olfactory functions. The bulk of the nasal mucosa is respiratory mucosa, which can adhere to dust and foreign substances. The mucosal epithelium was a pseudo lamellar columnar ciliated epithelium.

**Figure 1:**
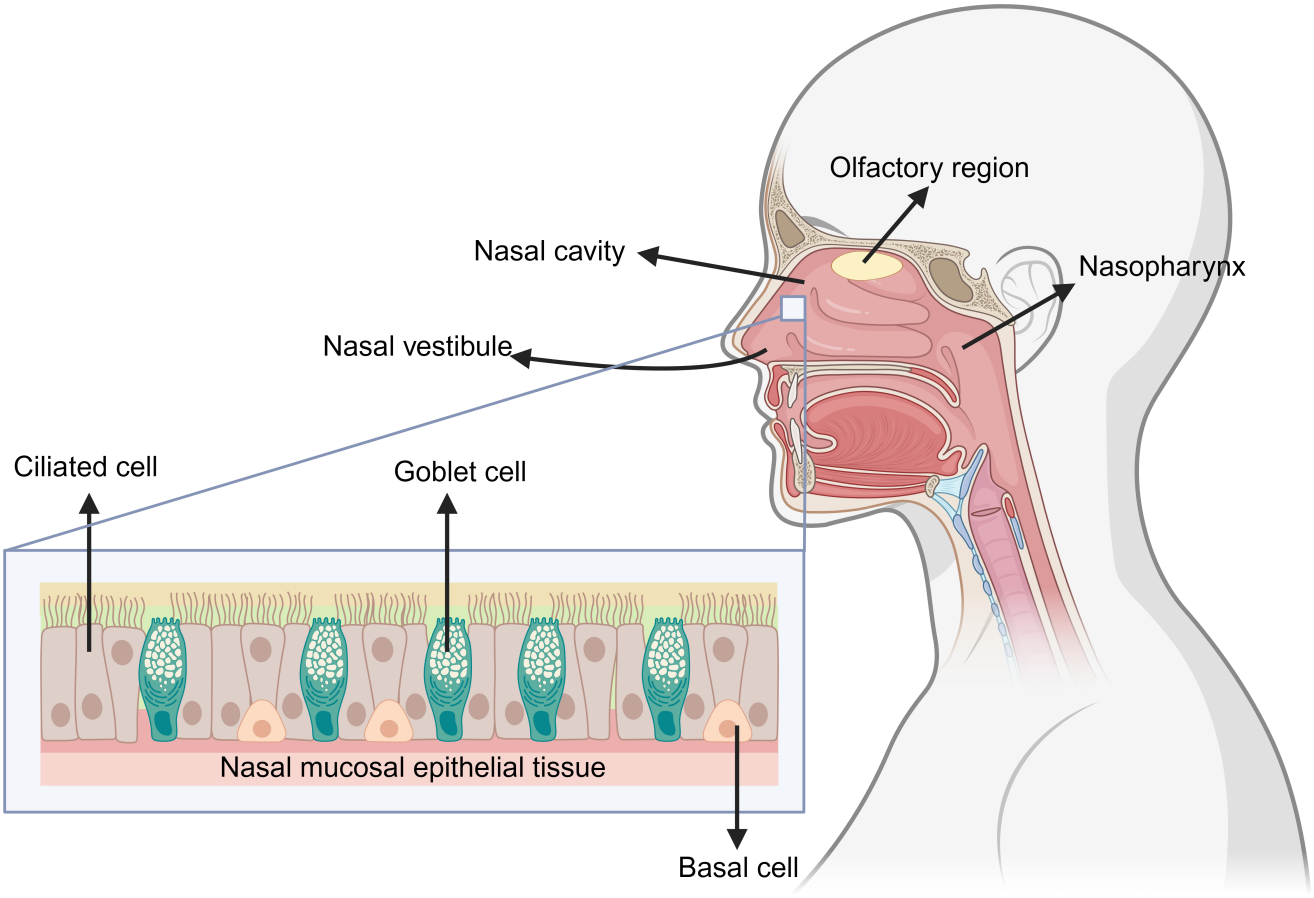
Structure of the nasal cavity. The nasal cavity is categorised into the right and left nasal passages by the nasal septum. Each nasal cavity is classified by the nasal threshold into the nasal vestibule and proper nasal cavity. The proper nasal cavity is covered by mucous membranes, which can be categorised into olfactory and respiratory mucous membranes, and respiratory mucous membranes account for most of the nasal mucous membranes. The nasal mucosal epithelium comprises ciliated goblets and basal cells.

**Figure 2:**
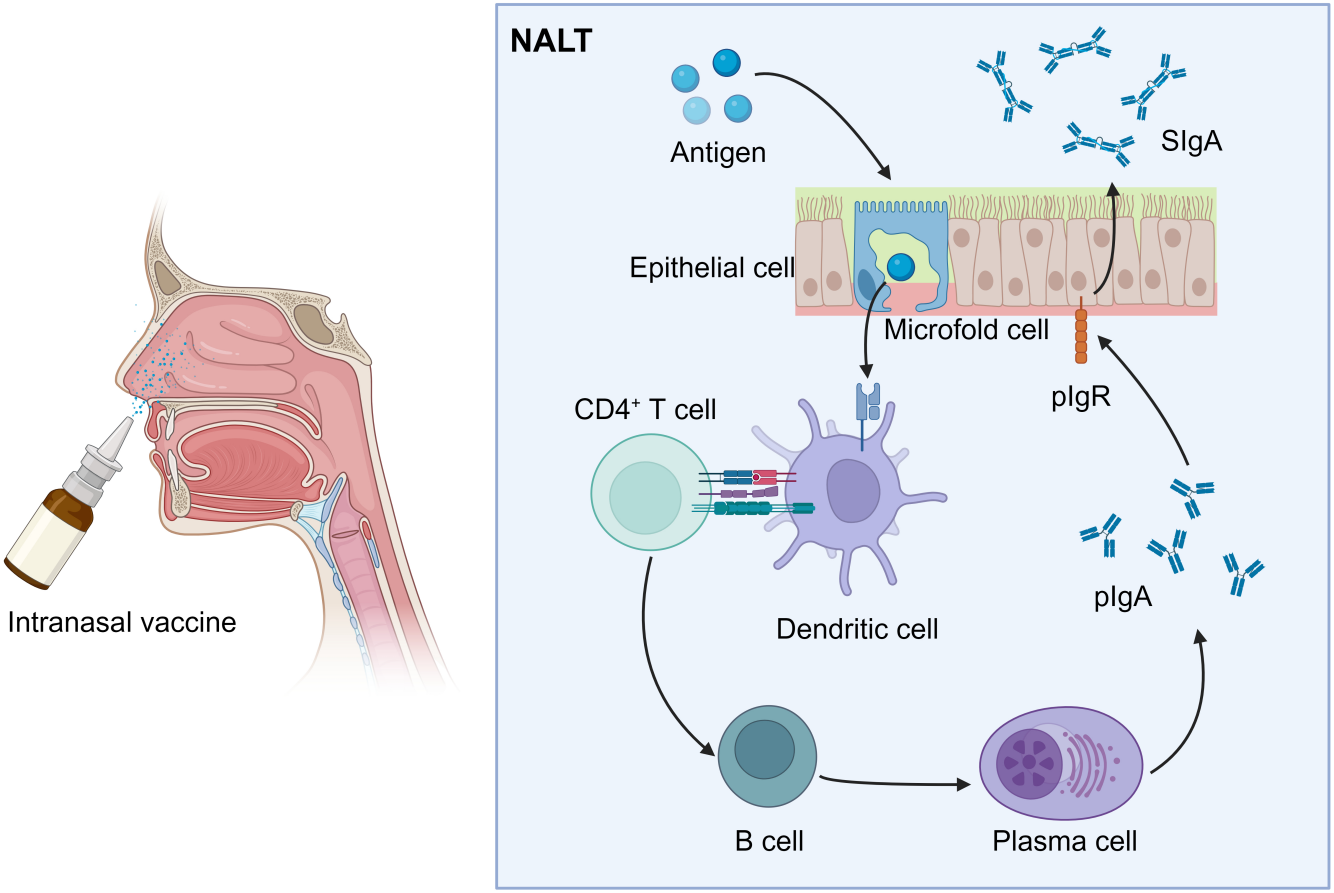
Mechanisms of IgA production by nasal mucosal immunity. When the virus antigen enters NALT, it is transported by microfold cells in the epithelial tissue, absorbed by antigen‐presenting cells such as dendritic cells and phagocytes, and presented to CD4^+^ T cells. CD4^+^ T cells promote the differentiation of B cells into plasma cells to produce pIgA and eventually form SIgA, the main effector of mucosal immunity, with the help of pIgR.

The nasal mucosal epithelial tissue was composed of ciliated, goblet and basal cells (Figure 1). Cilia and microvilli, which exhibit mucociliary clearance (MCC), are found on the surface of ciliated cells. The goblet cells release mucus. The nasal mucus is separated into the following two layers: the periciliary layer of mucin molecules and the mucus layer above it.^19^ When the virus enters the nasal cavity, the double layer of mucus and tightly bound ciliated goblet and basal cells are the first lines of defence. The MCC is another defence mechanism. When dust, viruses, particles and other foreign substances enter the nasal cavity and stick to the mucosa, tens of thousands of cilia of the mucosal epithelium move in unison to expel the mucus and the substances attached to it into the nasopharynx and throat, and subsequently enter the stomach through the oesophagus to be decomposed.^20^ The mucosal immune response is activated when a virus penetrates the defence barrier. The main function of mucosal immunity is to remove pathogens that invade the body through the mucosal surface. Nasopharynx‐associated lymphoid tissue (NALT) is the site of the nasal mucosal immune response. NALT is the Waldeyer's ring located in the pharynx, consisting of palatine tonsils, adenoids, tubular tonsils and lingual tonsils.^20^ Its interior comprises T cells, B cells and antigen‐presenting cells (APCs) such as dendritic cells and macrophages, which are covered with a layer of epithelial cells containing microfold cells (M cells).^14^ M cells play a key role in triggering mucosal immunity and are key targets for viral antigens to cross the epithelial cell barrier. When the virus reaches the NALT, the soluble antigen of the virus can be recognised directly by the APCs, while particulate antigens need to be transported by the M cells, then absorbed by the APCs and presented to the CD4^+^ T cells in the mucosal lymphoid tissue.^21^, ^22^ Subsequently, CD4^+^ T cells interact with B cells, inducing B cells to differentiate into plasma cells capable of secreting polymeric IgA (pIgA), which passes through mucosal epithelial cells to the mucosal surface under the action of polymeric Ig receptor (pIgR), and finally forms secretory IgA (SIgA)^14^ (Figure 2). It is a primary effector of mucosal immunity.^23^ IgA molecules in the blood exist as monomers, whereas SIgA is a dimer formed by connecting two IgA molecules that are involved in the neutralisation and elimination of invading infections. SIgA works in three main ways: (1) preventing the attachment of foreign pathogens to mucosal epithelial cells, known as immune exclusion; (2) inhibiting the replication, transcription and assembly of intracellular viruses, or neutralising the activity of toxins, known as intracellular neutralisation; and (3) using immunoglobulin to excrete antigens from mucosal lamina propria to the mucosal surface of epithelial cells, known as antigenic excretion.^24^, ^25^

## Variants of SARS‐CoV‐2

**Figure 3:**
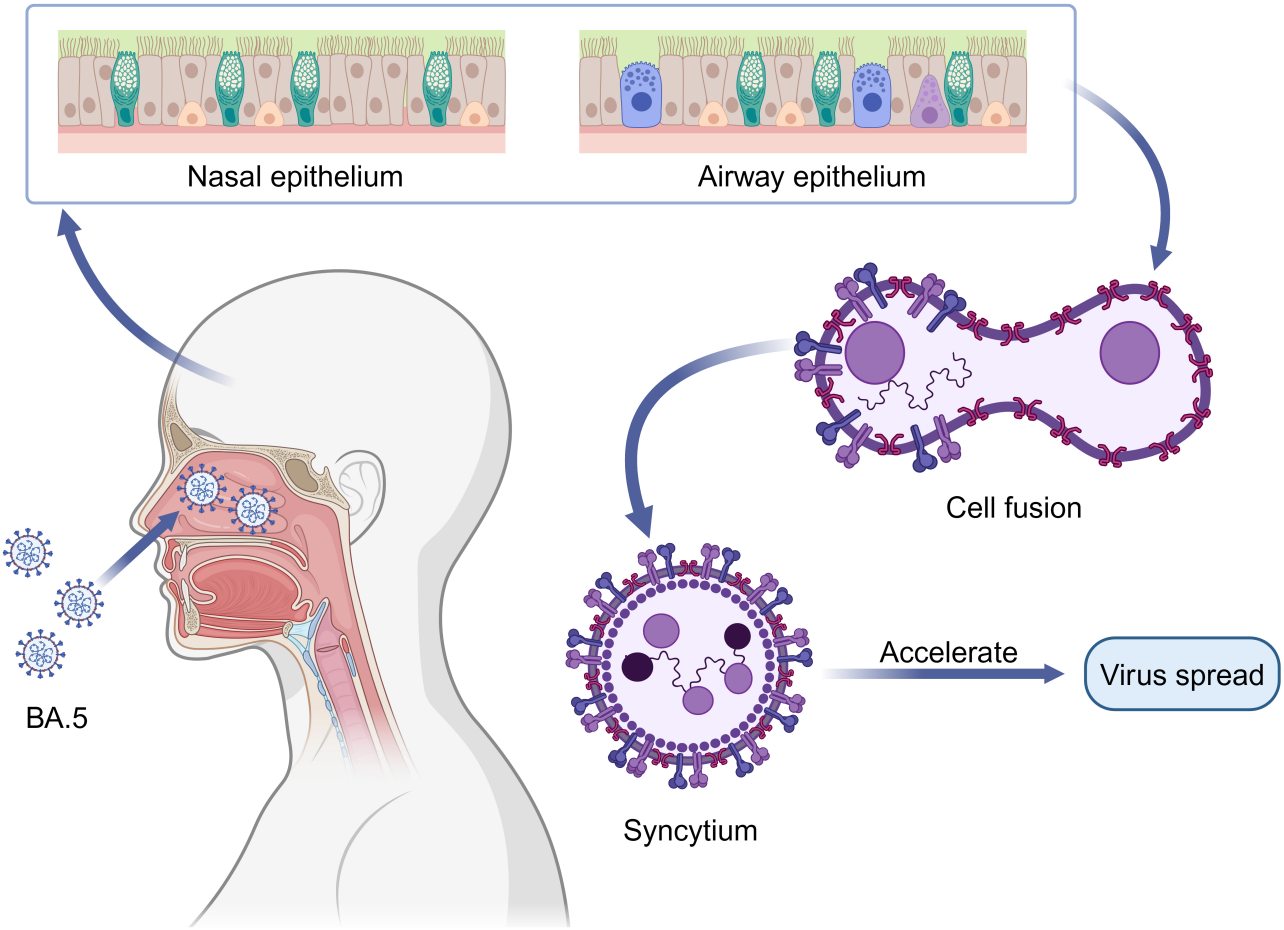
BA.5 promotes viral transmission by enhancing syncytial formation. The BA.5 subvariant of Omicron can induce the fusion of nasal mucosa and airway cells to form a syncytium after invading the human body, thereby promoting the spread of the virus.

Coronavirus disease 2019 (COVID‐19) is caused by a novel type of coronavirus known as SARS‐CoV‐2.^26^ According to the WHO (as of March 31, 2024), more than 700 million people have been diagnosed with COVID‐19, and more than 7 million have died of the disease.^27^ This seems to be one of the toughest challenges we have ever faced. SARS‐CoV‐2, similar to all viruses, mutates over time. On May 31, 2021, the WHO announced that the name of the SARS‐CoV‐2 variant would be changed to the Greek alphabet, although this nomenclature would not replace the original scientific nomenclature.^28^ In 2020, Alpha, Beta, Gamma, Delta and other mutants were discovered. The Omicron mutant, identified in November 2021, became the current pandemic strain, whereas the four preceding versions became historical epidemics.^29^ Omicron is a faster and more insidious form of SARS‐CoV‐2 than earlier circulating variants.^30^ Furthermore, Omicron has many sub‐variant strains, including BA.1, BA.2, BA.4 and BA.5, and each mutant has a unique set of traits. BA.5 is more infectious and transmissible than the earlier variants because it enables human nasal mucosal and airway cells to form syncytial cells to promote the spread of the virus^31^ (Figure 3). However, attention has recently been focused on the new circulating strains XBB and BQ and their subvariants because of their surprising immune escape ability. BQ.1 and BQ.1.1 evolved from BA.5. XBB, XBB.1 and XBB.1.5 are recombination products of the two BA.2 pedigree variants. They have strong antibody evasion ability and can effectively evade mRNA vaccines or humoral immunity induced by natural infection.^32^ BQ.1.1 is approximately six times more resistant to serum neutralisation than BA.5; however, XBB.1 is 63 and 49 times more resistant than BA.2 and BA.4/5, respectively.^33^ Additionally, current drugs, such as imdevimab‐casirivimab, tixagevimab‐cilgavimab, sotrovimab and bebatelovimab, may not be effective against either BQ.1.1 or XBB.^34^ Currently, the XBB series strains have the strongest antibody escape ability, far exceeding BA.5, and have reached or even exceeded the escape degree of SARS‐CoV‐1.^35^ Recently, XBB.1.5 infections in the United States have increased dramatically, quickly surpassing BQ.1.1 and other XBB subtypes. On February 18, 2023, the COVID‐19 strain surveillance data from the Centers for Disease Control and Prevention revealed that XBB.1.5 mutants accounted for 80.2% of the newly detected strains in the United States.^36^ Why does the XBB.1.5 have such strong propagation? This is because this strain has a higher affinity for human ACE2 receptors and a neutralising antibody escape capability similar to XBB.1.^37^ ACE2 is a receptor required for SARS‐CoV‐2 to invade host cells. XBB.1.5 is not the final Omicron variation. Therefore, for public health, it is necessary to identify strategies to combat SARS‐CoV‐2 infection.

## Mechanisms of SARS‐CoV‐2 infection in nasal epithelial cells

The transmission modes of SARS‐CoV‐2 are primarily respiratory and contact transmission. Virus‐carrying aerosols are the primary mode of transmission of SARS‐CoV‐2 over short and long distances.^38^, ^39^ People exhale aerosols when they breathe, talk, cough and sneeze, and the expelled aerosols can be transmitted through air.^40^, ^41^ We can become infected when we breathe in virus‐laden aerosols or touch our nose, mouth, or eyes after contact with an object infected with the virus.^3^ These viral aerosols can remain infectious in the air for more than 3 h,^42^, ^43^ or even for several days when attached to objects.^43^, ^44^, ^45^

**Figure 4:**
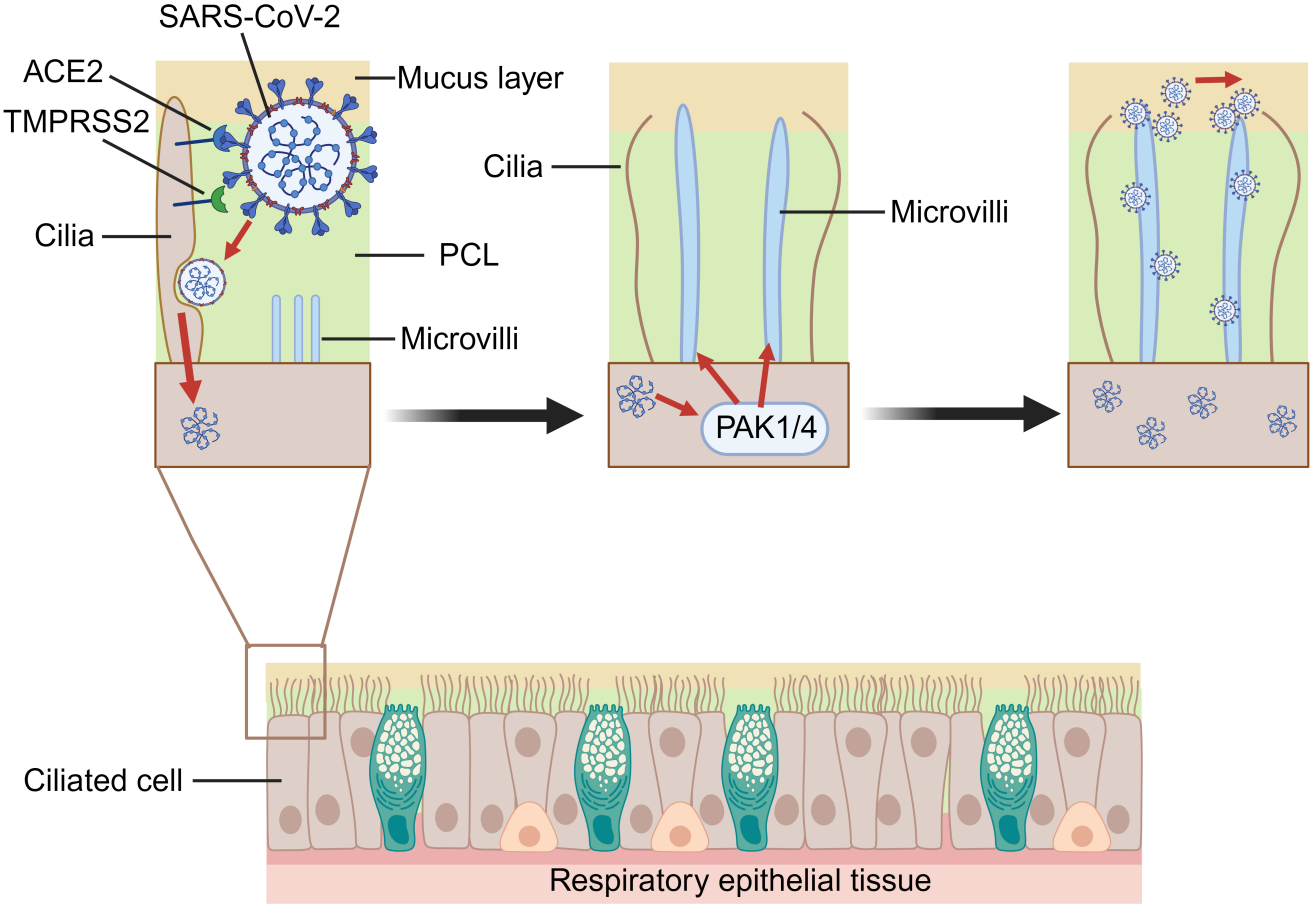
Mechanisms of virus infection in respiratory epithelial cells. SARS‐CoV‐2 first infects the respiratory ciliary cells. Viral invasion requires binding to ACE2 receptors on the surface of host cells. TMPRSS2 plays an assist role in the process of invasion. The invading virus activates p21‐activated kinases 1 and 4 (PAK1/4) to promote microvilli elongation. The newly generated virus is subsequently delivered through the microvilli to the mucus layer, allowing rapid viral spread. PCL, periciliary layer.

In 2020, goblet and ciliated cells in the nasal airway were identified as possible initial infection sites for SARS‐CoV‐2 owing to their high expression of ACE2 and TMPRSS2.^5^ The spike protein of SARS‐CoV‐2 binds to the ACE2 receptor, which acts as a door lock for the virus to enter human cells. TMPRSS2 assists in viral invasion by activating the SARS‐CoV‐2 spike protein and facilitating its entry into the cells. Currently, we have a better understanding of how SARS‐CoV‐2 infects respiratory epithelial cells. SARS‐CoV‐2 first infects respiratory ciliated cells; if the cilia are removed, infection with SARS‐CoV‐2 and other respiratory viruses can be prevented.^46^ Invading viruses simultaneously activate kinases in cells that promote cytoskeleton formation and deliver newly generated viruses to the mucous layer via highly extended microvillus structures, thereby improving their ability to spread^46^ (Figure 4). The nasal viral load is closely related to the severity of COVID‐19 symptoms and the transmission capacity of SARS‐CoV‐2.^47^ These findings suggest that the nasal cavity plays a vital role in preventing SARS‐CoV‐2 infections.

## Nasal saline cleaning

The strong immune escape ability of the Omicron‐variant strains significantly increases the risk of reinfection and infection after vaccination; however, reducing the nasal viral load and enhancing the self‐cleaning and immunity of the nasal mucosa can reduce the risk of SARS‐CoV‐2 infection. Therefore, strengthening nasal cleaning care and enhancing nasal mucosal immunity is particularly important to preventing and treating SARS‐CoV‐2. Notably, saline cleaning of the nasal cavity can remove dust particles and air pollutants from the nasal cavity, improve the oscillating function of mucociliary cilia, reduce mucociliary edema, promote local blood circulation, and enhance the mucociliary cleaning function.^48^ Simultaneously, saline increases the frequency of ciliary oscillations and reduces the production of local inflammatory mediators.^49^ Currently, many studies have shown that nasal cleaning can prevent COVID‐19 to a certain extent and reduce the symptoms of SARS‐CoV‐2 infection.^7^, ^50^, ^51^, ^52^ A study found that patients who did not receive nasal irrigation had an overall risk of hospitalisation or death 8.57 times greater than those who did.^52^ Moreover, saline nasal irrigation can shorten the duration of symptoms in patients^51^ and can even reduce the duration of the disease by 1.9–2.5 days when combined with gargling.^50^, ^53^

## IM vaccine

After contacting SARS‐CoV‐2, individual symptoms may be different. Most people infected have mild or moderate symptoms, such as fever, cough, tiredness, and loss of smell or taste and in a very small number, severe symptoms, including difficulty breathing, chest pain and loss of speech or movement.^2^ Although most individuals have mild symptoms, damage to the human body cannot be ignored. Infection with SARS‐CoV‐2, even in mild cases, can alter an individual's immune status. Men trigger a stronger inflammatory response to other viruses, leading to more pronounced functional changes in their immune system, even long after recovery.^54^ Additionally, SARS‐CoV‐2 infection can cause myocardial damage, including myocarditis, types 1 and 2 myocardial infarctions, and multisystemic inflammatory syndrome in children and adults.^55^

Injectable vaccines are widely used globally to combat SARS‐CoV‐2 infection. At present, IM vaccines against SARS‐CoV‐2, such as live attenuated vaccine, inactivated vaccine, protein subunit vaccine, viral vector vaccine, DNA vaccine and mRNA vaccine, have been widely developed and used.^56^, ^57^ IM vaccines can prevent SARS‐CoV‐2 infection to some extent and reduce the severity of the viral infection,^58^, ^59^ with a good efficacy of 66.7–94.6%.^60^ Moreover, studies have shown that IM vaccines can reduce the likelihood of Long COVID in patients with COVID‐19 and improve their long‐term quality of life.^61^, ^62^ Long COVID is widely defined as persistent symptoms after the initial infection with SARS‐CoV‐2 that last for more than 4 weeks after the initial stage of infection and progress or worsen over time, with severe and life‐threatening events likely to occur months or years after infection.^63^ At the beginning of the outbreak of SARS‐CoV‐2, natural infection or vaccination can provide the body with strong short‐term immunity against later variant strains.^64^ That does not mean people will not be reinfected because SARS‐CoV‐2 variants are so vast, and based on the second section above, new variants are likely to have greater immune escape. Moreover, the antibodies produced in the body after the initial infection are not permanent, and the titre of the antibodies will gradually weaken over time.^65^, ^66^ Multiple lines of evidence have shown that natural exposure to SARS‐CoV‐2 does not confer complete immunity.^67^, ^68^, ^69^, ^70^, ^71^, ^72^ Secondary infection can occur within 5–12 months of the initial infection.^73^ Vaccines provide only temporary protection, and their effectiveness declines over time.^74^, ^75^, ^76^ For the Omicron variant and the Delta variant, the vaccine efficacy of the former is generally lower than that of the latter.^77^ The effectiveness of two doses of the BNT162b2 vaccine against the Omicron variant was only 8.8% after 25 weeks.^77^ Moreover, IM vaccines are not good enough at stopping the spread of the virus.^14^, ^78^, ^79^ At present, most of the IM vaccines developed in the world can only induce systemic immune response and cannot induce mucosal immune response.^10^, ^11^, ^12^, ^13^, ^14^, ^15^, ^80^, ^81^ However, as the first stop of virus invasion, mucosal immunity in the nasal cavity is crucial for neutralising the virus, preventing further transmission of the virus down the respiratory tract, and preventing disease deterioration. IM vaccines induce systemic immune responses dominated by IgG. However, IgG antibodies in the circulatory system decay quickly and cannot be distributed to the nasal cavity and respiratory mucosa in time, making it difficult to play an effective role in preventing infection.^16^, ^82^ Only a very high serum IgG titre can achieve complete protection of the nasal cavity.^82^ Previous experiments on animals with IM vaccines have found that although the vaccine protects these animals well, the virus can still be detected in their nasal cavities.^78^, ^79^

## IN vaccine

Intranasal vaccines may change that. IN vaccines can not only induce a systemic immune response but also induce a mucosal immune response in nasal and respiratory tissues dominated by IgA antibodies, which can quickly prevent further invasion of the virus when it enters the human body.^16^, ^17^ IgA can neutralise SARS‐CoV‐2.^83^, ^84^, ^85^ SIgA antibodies, as a dimeric form of IgA, have higher viral‐neutralising activity than IgG antibodies.^86^, ^87^

Storage and transportation of a vast majority of currently approved IM vaccines against SARS‐CoV‐2 require cryogenic conditions.^80^ Temperature control is critical to maintaining the efficacy of the vaccine. Consequently, the availability of infrastructure and technology limits vaccination coverage in remote and poor areas. However, most IN vaccines currently in development are designed to be thermostable, with less stringent temperature requirements.^80^ For developing countries with underdeveloped medical conditions, the intranasal mucosal vaccine is more conducive to promotion. The nasal inhalation vaccine has low requirements for the injection personnel, is simple to operate, does not require the presence of medical personnel with professional medical training, and even the vaccinator can independently complete the vaccination at any time and anywhere. This needle‐free vaccine cannot only save human resources but also effectively reduce the recycling cost of medical waste such as needles and syringes and its secondary pollution to the environment. Furthermore, needle‐free vaccination can effectively improve the compliance of vaccinators, especially children, and can avoid local adverse reactions caused by intramuscular injection.

Some people may experience symptoms of anosmia after infection with SARS‐CoV‐2, which seriously impacts their daily lives, and its duration is highly uncertain. Although common respiratory viral infections can cause anosmia, SARS‐CoV‐2 infections are associated with a significantly higher proportion of anosmia.^88^ The principle of influenza virus‐induced anosmia may be divided into two types: cell death caused by direct invasion of cells by the virus and olfactory sensory cells infected by the virus secrete a large number of proinflammatory cytokines so that they are cleared by inflammatory cells.^89^ Intranasal influenza vaccination has been found to attenuate the production of influenza virus mRNA and the upregulation of inflammatory mediators in the olfactory bulb.^90^ However, anosmia caused by SARS‐CoV‐2 infection does not appear to follow the same principle because ACE2 or TMPRSS2 is not expressed in olfactory sensory nerve cells, implying that SARS‐CoV‐2 cannot invade olfactory sensory nerve cells at all.^91^, ^92^ In fact, the olfactory epithelial barrier supporting cells are the main targets of SARS‐CoV‐2 infection, and the damage to olfactory sensory nerve cells is due to severe dysfunction of the immune system in the olfactory epithelium due to viral infection, leading to a persistent and uncontrolled inflammatory response in the olfactory epithelium.^93^ The olfactory disorder caused by SARS‐CoV‐2 may be related to mucosal immune deficiency.^94^ IN vaccine can enhance the function of local immunity and mucosal immunity in human body, and may be effective for the treatment of anosmia caused by SARS‐CoV‐2.^95^ However, objective data on either intranasal influenza vaccines or SARS‐CoV‐2 vaccines, to further test their ability to prevent anosmia, are still lacking.

Currently, more than 10 kinds of IN vaccines have entered clinical trials worldwide.^8^ Innovative and forward‐looking research on these vaccines will significantly help to improve their protection. Additionally, considering the complementary immune protection mechanism of the nasal spray and intramuscular injection vaccines, the nasal spray vaccine will become a crucial complementary point for the SARS‐CoV‐2 vaccine and a turning point in the fight against SARS‐CoV‐2.

### Viral vector IN vaccine

Adenovirus (Ad) has attracted much attention since its discovery because of its strong immunogenicity and good safety.^96^ Ad vaccine, as a relatively mature carrier vaccine platform, has been widely developed to combat SARS‐CoV‐2. Adenovirus serotype 5 (Ad5), one of the most commonly used Ad vectors, has also been widely used in the development of IN vaccines. The recombinant Ad5 vector vaccine Ad5.SARS‐CoV‐2‐S1, which encodes the S1 subunit antigen gene of SARS‐CoV‐2, has been shown to induce strong and long‐term specific antibody and cellular immune responses in mice.^97^ Another Ad5 vector vaccine, Ad5‐S‐nb2, can induce systemic and pulmonary antibody responses in rhesus monkey experiments, which can effectively and quickly clear the virus entering the respiratory tract and lungs.^98^

A vaccine developed by Bharat Biotech in India, ChAd SARS‐CoV‐2‐S (BBV154), is a recombinant chimpanzee adenovirus vector vaccine that induces long‐term, high levels of IgA with a single intranasal dose.^99^, ^100^ Moreover, it showed strong anti‐infection ability in protecting the upper and lower respiratory tract against SARS‐CoV‐2 variants B.1.351 (Beta) and B.1.1.28 (Gamma), compared with the lower protection of the upper respiratory tract by intramuscular injection.^99^, ^100^ At present, this vaccine has completed the clinical study of Phase I to Phase III.^8^

Another chimpanzee adenovirus vector vaccine, ChAdOx 1 nCoV‐19 (AZD1222), was previously approved for use as an IM vaccine in the United Kingdom on December 30, 2020.^101^ At present, the University of Oxford is already developing the nasal use of the vaccine and has entered Phase I clinical trials.^102^ Compared with other respiratory mucosal vaccines, vaccines based on Ad5 and chimpanzee Ad have better efficacy and safety.^103^ IN vaccines based on chimpanzee Ad are likely to be more effective than those based on human Ad, thus can carry on further clinical research and development.^104^

There are also vaccines based on influenza viruses. CA4‐dNS1‐nCoV‐RBD, an IN vaccine based on live attenuated influenza virus vector, not only induces a rapid and durable respiratory immune response against the prototype, Beta and Omicron variants of SARS‐CoV‐2 but also has a certain influenza virus protection effect, providing a coping method for the possible coexistence of SARS‐CoV‐2 and influenza in the future.^105^ Subsequent human trials established that the vaccine had a good safety profile, with an overall incidence of adverse reactions of only 19%.^106^ It is the world's first IN vaccine to enter Phase III clinical trials and has already been approved for use in China in December 2022 under the name Pneucolin.^107^

### Live attenuated IN vaccine

Live attenuated vaccine refers to the pathogen after various treatments, will be inoculated into the human body, can imitate natural infection and induce the body's immune response, but will not cause disease. For influenza viruses, live attenuated vaccines induce an immune response that more closely resembles natural immunity than other types of vaccines.^108^, ^109^

MV‐014‐212, a recombinant, attenuated live IN vaccine, can produce systemic and mucosal immunity after a single intranasal dose in animal experiments, induce mucosal IgA and serum IgG, has good resistance to Alpha, Beta and Delta variants, and has now entered Phase I clinical trials.^110^ Another live attenuated vaccine, COVI‐VAC, is also in clinical trials.^111^

### Protein subunit IN vaccine

The protein subunit vaccine platform is widely used due to its high safety and low cost.^112^, ^113^ The spike protein of SARS‐CoV‐2 is the most important of its four structural proteins. spike protein has two subunits: S1 subunit and S2 subunit. The S1 subunit contains the receptor binding domain (RBD), which has the function of recognising host cell surface receptors and mediating virus invasion into host cells. Therefore, the spike protein is a key target molecule for vaccine development.

Intravacc has developed an intranasal protein subunit vaccine containing a recombinant spike protein HexaPro based on a D614G mutation and based on outer membrane vesicles (OMV).^114^ Intranasal injection of the vaccine can induce a large number of IgA and IgG antibodies in the respiratory tract, effectively preventing virus replication, while intramuscular injection can only cause serum IgG reaction, and the vaccine has now entered Phase I clinical trials.^114^

Mambisa (CIGB‐669) is an intranasal protein subunit vaccine developed by the Cuba Center for Genetic Engineering and Biotechnology. It uses Hepatitis B virus core protein (AgnHB) as an immune vector and is effective in stimulating mucosal immune responses.^115^ The vaccine is currently in Phase I/II clinical trials to investigate its immunogenicity and safety in the population.^115^

ACM‐001, developed by ACM Biolabs, is an adjuvant booster vaccine against SARS‐CoV‐2 containing components from the β‐type SARS‐CoV‐2 spike protein and the immune stimulator CpG7909, which is currently in Phase I clinical trials.^116^

At present, there is no intranasal mRNA vaccine against SARS‐CoV‐2 in clinical trials, but earlier studies have found the feasibility of an intranasal mRNA vaccine against tuberculosis.^117^ Although mRNA vaccines have high storage temperature requirements, this problem can be improved by making certain chemical modifications to the mRNA molecules.^118^, ^119^ In view of the good safety and efficacy of the mRNA vaccine and its short production time, which can quickly respond to various SARS‐CoV‐2 mutants,^120^ researchers can pay more attention to the development of the intranasal mRNA vaccine. Furthermore, given the complementary characteristics of the intramuscular and IN vaccines in inducing immune responses, the IN vaccine as a supplement to the IM vaccine may be a more effective measure against various variants of SARS‐CoV‐2 in the future, which may be proven by relevant clinical trials.

The development of IN vaccines faces many challenges. First of all, as mentioned above, the nasal surface has a protective barrier of cilia and mucus to prevent the intrusion of foreign substances. Therefore, increasing the residence time of the vaccine in the nasal cavity so that the antigen can be fully absorbed is one of the difficulties that researchers need to consider. Second, IN vaccines often require vaccine adjuvants, and finding vaccine adjuvants that can maintain antigen stability under the premise of fully activating the immune system is also a challenge.^10^ Third, there are constraints on the size of particles ejected by IN vaccine delivery devices. Large particles tend to deposit in the front of the nasal cavity and can be easily exhaled or wiped off, while too small particles may pass directly through the nasal cavity and deposit in the lungs, leaving the upper respiratory tract unprotected.^121^ Finally, the safety of vaccines cannot be ignored. At present, the vast majority of IN vaccines are still in the preclinical or early clinical trial stage, so the safety of these vaccines in humans is not clear.

## Nasal spray

The spread of viral aerosols can be limited to some extent by wearing masks.^122^, ^123^ However, masks are no more than 70% effective in protecting the wearer.^124^ Considering the importance of preventing various SARS‐CoV‐2 variants in the future, it is necessary to develop new strategies for nasal protection. Recently, an “intranasal mask” may be a good way to deal with this problem. It is a nasal spray formulation consisting of a hydrogel and tiny vesicles containing specific viral receptors that, when sprayed into the nose, can rapidly change from liquid to gel‐like attachment to the lining of the nasal cavity.^125^ The hydrogel not only protects the nasal cavity from virus infection but also traps viral aerosols to prevent them from infecting the lungs, while receptors on the vesicles capture and inactivate the virus.^125^ This new nasal spray can compensate for the inadequate protection of current masks and further reduce the risk of SARS‐CoV‐2 infection.

## Harms of SARS‐CoV‐2 coexisting with influenza

As SARS‐CoV‐2 spreads worldwide, we should not only be vigilant about it but also take precautions against influenza viruses. Evidence exists that influenza infection promotes SARS‐CoV‐2 infection in human cells, and both may even promote infection. This mode of promotion manifested as enhanced ACE2 receptor expression.^126^, ^127^ The data demonstrated that the mRNA levels of ACE2 receptors in host cells increased by 2–3 times after infection with influenza and by 28 times in cases of coinfection with influenza and SARS‐CoV‐2.^127^ But other studies suggest otherwise. One study found that the risk of contracting SARS‐CoV‐2 after influenza infection was reduced by 58%.^128^ There is a mathematical model to support this conclusion. If one virus grows faster than the other, that virus will suppress the other virus.^129^ SARS‐CoV‐2 grows much more slowly than the influenza virus; therefore, it is easy to suppress SARS‐CoV‐2 if you are infected with the influenza virus first. However, if you are infected with SARS‐CoV‐2 first, co‐infection will occur much more easily.^130^ At present, it is not clear whether co‐infection will lead to more serious consequences for patients. Co‐infection will increase the severity and mortality of infected persons according to most studies.^131^, ^132^, ^133^, ^134^, ^135^ Co‐infection causes twice the mortality rate of patients infected with SARS‐CoV‐2 alone.^128^ However, some studies have suggested that co‐infection has no effect on the disease outcome of patients.^136^, ^137^ Therefore, more extensive and systematic testing of SARS‐CoV‐2 and influenza viruses is necessary, and vaccination is recommended to prevent co‐infection, given that co‐infection may be more harmful to the human body. Some intranasal influenza vaccines, such as Fluenz Tetra and FluMist Quadrivalent, have already been approved for use.^14^ Moreover, the previously mentioned CA4‐dNS1‐nCoV‐RBD IN vaccine can protect against both SARS‐CoV‐2 and influenza viruses. The coexistence of SARS‐CoV‐2 and influenza viruses may become more common in the future, and the use of IN vaccines based on influenza virus vectors may become more obvious.

By March 31, 2024, more than 7 million people had died of COVID‐19 globally since the WHO declared the outbreak a global pandemic on March 11, 2020.^27^, ^138^ However, the global death toll from COVID‐19 reduced to its lowest level in 4 years. On April 10, 2023, United States President Joe Biden ended the nation's state of emergency in response to the coronavirus.^139^ On the same day, the United States government announced that it would spend $5 billion to develop a new generation of COVID‐19 vaccines and treatments, with nasal vaccines being a major target.^140^ It can be found that the IN vaccine is a significant development direction for future vaccines, which has a non‐negligible role in preventing and treating COVID‐19. IN vaccine can be used as a supplement to the IM vaccine, integrating the advantages of both vaccines to achieve better immune effects.

## Conclusion

Although we can breathe a sigh of relief in the battle against SARS‐CoV‐2, COVID‐19 is still not a common cold, and its harm to the human body cannot be ignored. Therefore, more attention should be paid to the role of the nasal cavity in preventing SARS‐CoV‐2 infection. As IN vaccines can complement the shortcomings of IM vaccines regarding immune mechanisms, IN vaccines should be further studied. Future studies should consider the safety and efficacy of IN vaccines in large populations.

## Conflict of interest

The authors declare no conflict of interest.

## Author contributions

**Ge Jin:** Conceptualization; investigation; visualization; writing – original draft; writing – review and editing. **Runze Wang:** Conceptualization; writing – review and editing. **Yi Jin:** Conceptualization; investigation. **Yingqiu Song:** Conceptualization; project administration. **Tianlu Wang:** Funding acquisition; resources; supervision.
